# Physico-Chemical Properties and Phase Behaviour of Pyrrolidinium-Based Ionic Liquids

**DOI:** 10.3390/ijms11041825

**Published:** 2010-04-21

**Authors:** Urszula Domańska

**Affiliations:** Department of Physical Chemistry, Faculty of Chemistry, Warsaw University of Technology, Noakowskiego 3, 00-664 Warsaw, Poland; E-Mail: Ula@ch.pw.edu.pl; Tel.: +48-22-6213115; Fax: +48-22-6282741

**Keywords:** 1-alkyl-1-methylpyrrolidinium-based ionic liquids, (solid + liquid) phase equilibria, water, alcohols, correlation, differential scanning microcalorimetry

## Abstract

A review of the relevant literature on 1-alkyl-1-methylpyrrolidinium-based ionic liquids has been presented. The phase diagrams for the binary systems of {1-ethyl-1-methylpyrrolidinium trifluoromethanesulfonate (triflate) [EMPYR][CF_3_SO_3_] + water, or + 1-butanol} and for the binary systems of {1-propyl-1-methylpyrrolidinium trifluoromethanesulfonate (triflate) [PMPYR][CF_3_SO_3_] + water, or + an alcohol (1-butanol, 1-hexanol, 1-octanol, 1-decanol)} have been determined at atmospheric pressure using a dynamic method. The influence of alcohol chain length was discussed for the [PMPYR][CF_3_SO_3_]. A systematic decrease in the solubility was observed with an increase of the alkyl chain length of an alcohol. (Solid + liquid) phase equilibria with complete miscibility in the liquid phase region were observed for the systems involving water and alcohols. The solubility of the ionic liquid increases as the alkyl chain length on the pyrrolidinium cation increases. The correlation of the experimental data has been carried out using the Wilson, UNIQUAC and the NRTL equations. The phase diagrams reported here have been compared to the systems published earlier with the 1-alkyl-1-methylpyrrolidinium-based ionic liquids. The influence of the cation and anion on the phase behaviour has been discussed. The basic thermal properties of pure ILs, *i.e.*, melting temperature and the enthalpy of fusion, the solid-solid phase transition temperature and enthalpy have been measured using a differential scanning microcalorimetry technique.

## 1. Introduction

Thanks to their interesting properties ionic liquids (ILs) are driving new research in many fields because of the interest in finding alternatives for conventional organic solvents. Their specific properties of a broad temperature range of liquid phase, extremely low vapour pressure, air and moisture stability, and specific electrochemical properties make ILs potentially useful in many technological processes [1–11]. The pyrrolidinium-based ILs were used in the preparation of a novel class of polymer electrolytes based on polymer-ionic liquid gels, which can be applied in electrochemical devices, especially in chemical capacitors [1–3]. They showed excellent performance as mechanically and electrochemically stable polymer matrixes for polymer electrolyte compositions with a wide electrochemical stability window (7.0V) [4]. The electrochemical reduction of palladium halide complexes, of nanocrystalline ZnO films and of tantalum and polycrystalline gold substrates using 1-butyl-1-methylpyrrolidinium bis(trifluoromethylsulfonyl)imide, [BMPYR][NTf_2_], was presented as well [5–7]. The conformational structure of the pyrrolidinium-based ionic liquid [BMPYR][NTf_2_] is well known [8] and is responsible for the interactions in the solutions. The pyrrolidinium-based IL was found to show a high ability to aggregate in aqueous solution, demonstrating its potential applicability as surfactant [9]. 1-Butyl-1-methylpyrrolidinium triflate was used in the catalytic processes with good economy, simplicity and as a recyclable medium [10]. The use of chromatographic and spectroscopic methods to establish the fundamental physico-chemical properties of over 200 ILs, including [BMPYR][NTf_2_], were described [11].

During the last ten years, measurements of the thermophysical and thermodynamic properties of ILs have increased remarkably, but they are not exhaustive enough for many new projects. Knowledge of the phase equilibria (vapour + liquid phase equilibrium, VLE, liquid + liquid phase equilibrium, LLE and solid + liquid phase equilibrium, SLE) is important if ILs are to be considered as solvents in technological processes [12–22]. The critical properties of ILs, including the pyrrolidinium-based ILs, and calculated by group contribution were presented by Valderrama and Rojas [12]. The first probe of the distillation of some ILs, including [BMPYR][NTf_2_], even though they exert no measurable vapour pressure, was made in a Kugelrohr apparatus at 573 K and at 6–8 mbar pressure [13]. The VLE data for [BMPYR][NTf_2_] with alkanes and alkenes showed the possible use of this IL in the alkane/alkene separation process [14,15]. The mod. UNIFAC (Dortmund) parameters were developed from VLE, activity coefficients at infinite dilution and excess enthalpies and were used in the prediction of the separation processes [14,15]. The heat capacities and heats of solution of 1-propyl-1-methylpyrrolidine bis(trifluoromethylsulfonyl)imide were measured in water, methanol and acetonitrile [16]. The densities of pyrrolidinium–based ionic liquids over a wide range of temperatures were measured and calculated using the Ye and Shreeve group contribution method [14,15,17]. Essentially, all of the solvents available for chemical engineering processes to control reactions, perform separations and process materials are usually common liquids such as toluene, benzene, acetonitrile, ethanol and water. The ionic liquids have a chance to revolutionize the way new technology is planned. After years of measurements of phase equilibria and of activity coefficients at infinite dilution we can conclude that the pyrrolidinium-based ionic liquids are very promising [14,15,18–22]. In the process of desulfurization of oils using ionic liquids (extraction of dibenzothiophene from dodecane using ILs), the pyrrolidinium-based ILs have shown the same selectivities as popular imidazolium-based ionic liquids, but worse than pyridinium-based ILs [19]. Recently, [BMPYR][NTf_2_] was described as a suitable solvent for extraction of aromatic hydrocarbons (benzene, toluene and ethylbenzene) from aliphatic hydrocarbons (heptane) [20]. The selectivity of a particular IL can be calculated for the different separation problems from measurements of the activity coefficient at infinite dilution [14,15,21,22]. The activity coefficient for various solutes (alkanes, alkenes, cycloalkanes, aromatics, alcohols, ketones, esters, ethers and water) were measured in the pyrrolidinium-based ILs: [BMPYR][NTf_2_] [14,15,21], [HMPYR][NTf_2_] [15], [OMPYR][NTf_2_] [15], and 1-butyl-1-methyl-pyrrolidinium triflate [BMPYR][CF_3_SO_3_] [22]. An entrainer should show a high selectivity at infinite dilution, a low viscosity, a high flash point, a low melting point and a boiling point higher that those of the separated components. Furthermore, it should be non-toxic, non-flammable, non-corrosive and recyclable. The pyrrolidinium-based ILs were found to be suitable as entrainers for the separation of aliphatic hydrocarbons from aromatic hydrocarbons, and of aromatic sulfur compounds from aliphatic hydrocarbons [20–22]. Repeated [BMPYR][CF_3_SO_3_] showed higher selectivity in the separation of aliphatic hydrocarbons from aromatic hydrocarbons than the popular imidazolium ionic liquid [BMIM][CF_3_SO_3_] [22]. On the other hand, the triflate anion showed much better selectivity than the bis(trifluoromethylsulfonyl)imide anion with the same cations in the different separation processes as for the examples mentioned above and alkane/thiophene [22].

Recent works have shown that some pyrrolidinium-based ILs have the potential to be good solvents for separating organic liquids using solvent extraction or extractive distillation processes with water [23,24]. In particular, the use of ILs with lower mutual solubilities with water as biphasic extraction media for the removal of organic compounds from water which is useful for industrial processes. The LLE of [PMPYR][NTf_2_] and [BMPYR][NTf_2_] with water were measured and predicted by the COSMO-RS [23,24]. The hydrophobic pyrrolidinium-based ILs have shown better solubility in water than piperidinium-based ILs and worse solubility in water than imidazolium-based and pyridinium-based ILs. Furthermore, the hydrophobicity of the alkyl-pyrrolidinium-based ILs increases when the alkyl chain length on the cation increases [24]. It is however the thermodynamic information, which reflects how the different IL interact with water, that is crucial in assessing its usefulness and allows one to predict the better and more efficient IL.

Recently, the mutual solubility of a pyrrolidinium-based IL, namely 1-butyl-1-methylpyrrolidinium triflate [BMPYR][CF_3_SO_3_], in hydrocarbons (hexane, heptane, cyclohexane, benzene, toluene) was measured in our laboratory [25]. It was shown using phase equilibria study of binary systems that aliphatic hydrocarbons are weakly soluble in [BMPYR][CF_3_SO_3_], whereas aromatic compounds are very soluble in this IL [25]. These are however, typical properties of many other ILs [26–29]. Phase equilibria results are also important in expanding our knowledge about the nature of ILs and in assisting in the systematic study of their thermodynamic properties. These results are also a good indication that the ILs involved will show good extraction properties. It was found that the investigated ILs could be very good entrainers for the separation of sulfur compounds from alkanes, as thiophene was completely soluble over a wide range of temperature [28]. Generally, the selectivity for the separation of aromatic hydrocarbons from the aliphatic hydrocarbons decreases with increasing length of the alkyl chain on the imidazolium, pyridinium, or pyrrolidinium cation, or on the anion of the IL.

The phase diagram of the 1-ethyl-1-methylpyrrolidinium bis(trifluoremethylsulfonyl)imide, [EMPYR][NTf_2_] with benzene was presented as an eutectic system with the miscibility gap in the liquid phase at the solvent rich phase [29].

One of the aims of this work was to measure solubilities of two pyrrolidinium-based ILs: 1-ethyl-1-methylpyrrolidinium trifluoromethanesulfonate (triflate) [EMPYR][CF_3_SO_3_] in water, or in 1-butanol} and of 1-propyl-1-methylpyrrolidinium trifluoromethanesulfonate (triflate) [PMPYR][CF_3_SO_3_] in water, or in an alcohol (1-butanol, 1-hexanol, 1-octanol, 1-decanol)} at atmospheric pressure using a dynamic method. The investigation also includes a discussion of the effect of the alkyl chain length of the alcohol and the alkyl chain length on the pyrrolidinium ring on solubility.

## 2. Results and Discussion

The phase diagrams of new binary {ionic liquid + water, or + an alcohol} systems were determined. The structures of the ILs are presented in Table 1. A brief thermophysical characterization of the two ILs is presented in Table 2.

**Figure 1.**
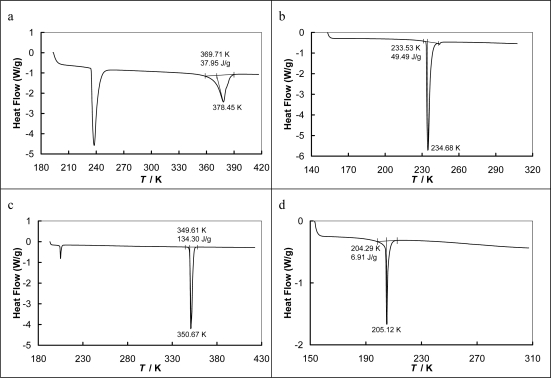
The DSC diagrams for: (a) [EMPYR][CF_3_SO_3_], the whole range of temperatures; (b) [EMPYR][CF_3_SO_3_], the lower temperature’s peak; (c) [PMPYR][CF_3_SO_3_], the whole range of temperatures; [PMPYR][CF_3_SO_3_], the lower temperature’s peak.

The basic thermal properties of the ionic liquids *i.e.*, temperature of fusion (*T*_fus_), enthalpy of fusion (Δ_fus_*H*), (solid-solid) phase transition temperature (*T*_tr_) and the enthalpy change of (solid-solid) phase transition (Δ_tr_*H*) have been measured. The enthalpy of melting of [EMPYR][CF_3_SO_3_] was found to be very small (9.99 ± 0.05 kJ·mol^−1^) in comparison with [PMPYR][CF_3_SO_3_] (37.24 ± 0.05 kJ·mol^−1^). This anomalous result can be explained by the second peak at DSC diagram for the (solid-solid) phase transition at a very low temperature (234.15 K) and with high enthalpy of phase transition (see Figure 1). The value of the enthalpy of the (solid-solid) phase transition (13.03 ± 0.05 kJ·mol^−1^) is much higher than the enthalpy of melting. The value of heat capacity changes during the melting point (Δ_fus_*C*p) was calculated from summarized values of Δ_fus_*H* and Δ_tr_*H*. The solubilities are the results of different interactions between the IL and water, or an alcohol. In this work the interaction may be due to the hydrogen bonding between pyrrolidinium ring of the cation and/or fluorine and oxygen atoms of the anion of the IL and of polar groups of the solvent. Unfortunately, both investigated ILs are solids at room temperature. It is known from the fundamental SLE discussion that the substance with the lower melting temperature and the lower enthalpy of melting is a better soluble in the same solvent.

**Figure 2.**
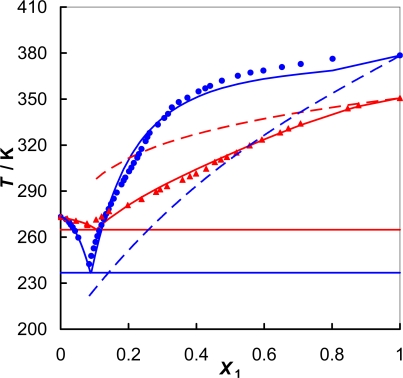
(Solid + liquid) phase equilibria of {IL + water}: (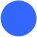) [EMPYR][CF_3_SO_3_]; (
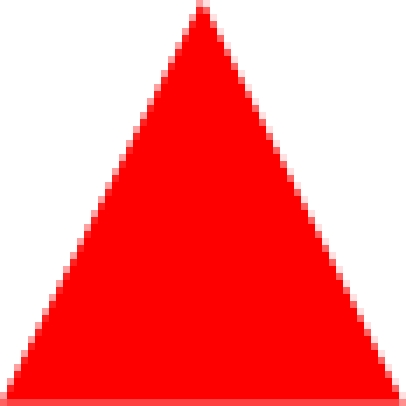
) [PMPYR][CF_3_SO_3_]. Solid lines designated by the Wilson equation. Dotted lines represent an ideal solubility.

The experimental (solid + liquid) phase equilibrium data for the systems {IL + water} are given in Figure 2. The experimental data, temperature, *T*^SLE^ *vs*. mole fraction of the IL, *x*_1_ are listed in Tables 3 and 4 for [EMPYR][CF_3_SO_3_] and [PMPYR][CF_3_SO_3_], respectively. The liquidus curves for the [EMPYR][CF_3_SO_3_] are almost the same as for [PMPYR][CF_3_SO_3_]. The [EMPYR][CF_3_SO_3_] shows lower solubility than [PMPYR][CF_3_SO_3_] because of its higher melting temperature. The experimental eutectic points are: for {[EMPYR][CF_3_SO_3_] + water}, *x*_1,e_ = 0.084, *T*_e_/K = 242.5; and for {[PMPYR][CF_3_SO_3_] + water}, *x*_1,e_ = 0.079, *T*_e_/K = 267.9. The eutectic points are at the similar compositions, but at different temperatures. The eutectic points are shifted strongly to the solvent rich side.

**Figure 3.**
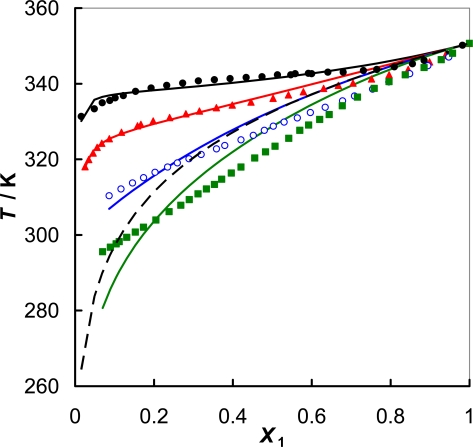
(Solid + liquid) phase equilibria of {[PMPYR][CF_3_SO_3_] + an alcohol}: (•) 1-decanol; (
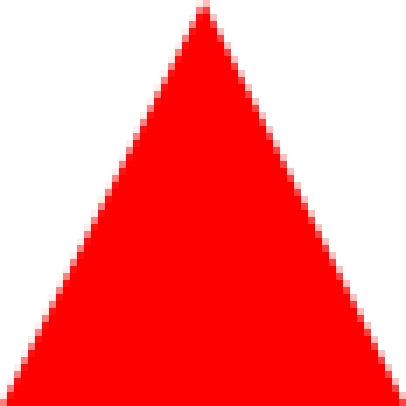
) 1-octanol; (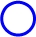) 1-hexanol; (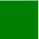) 1-butanol. Solid lines designated by the Wilson equation. Dotted line represents an ideal solubility.

On the basis of the phase diagram presented in Figure 3 for the {[PMPYR][CF_3_SO_3_] + an alcohol}, the following trends can be seen: for all the mixtures, simple eutectic systems were observed with complete miscibility in liquid phase; and the solubility of the IL in alcohols decreases as the length of the carbon chain of an alcohol increases; for alcohols the eutectic temperatures were below 290 K, the lowest temperature we could attain in these systems.

The comparison of SLE data for two investigated ILs (IL + 1-butanol) with the same anion [CF_3_SO_3_]^−^ but different cations ([EMPYR]^+^ and [PMPYR]^+^) shows the differences connected only with the melting temperature of the IL. The liquidus curve of [EMPYR][CF_3_SO_3_] in 1-butanol is at much higher temperature. It means that this IL is miscible with 1-butanol over the liquidus curve at a higher temperature. Thus the increasing of the alkane chain length at the pyrrolidinium ring decreases the melting temperature of the IL and increases the solubility in water and in alcohols. This is probably the influence of the more asymmetric cation of the [PMPYR][CF_3_SO_3_] and its influence on the interaction with a polar solvent. Furthermore, it was observed that the solubility of [EMPYR][CF_3_SO_3_] in water was lower than the ideal solubility (see activity coefficients *γ*_1_ > 1 in Table 3) but those of [PMPYR][CF_3_SO_3_] in water and in the shorter chain length alcohols was higher than the ideal solubility (see activity coefficients *γ*_1_ < 1 in Table 4). The solubility of [EMPYR][CF_3_SO_3_] is lower in water than in 1-butanol, and those of [PMPYR][CF_3_SO_3_] is higher in water than in 1-butanol. This is the evidence, that the more asymmetric cation of the [PMPYR][CF_3_SO_3_] increases the solubility in alcohol in comparison with water. It has to be the van der Waals interaction between the alkane chains of cation and that of an alcohol.

A simple eutectic system with complete miscibility in the liquid phase for binary systems {IL + water} was observed earlier for many ionic liquids, *i.e.*, for imidazolium-based ILs, 1-butyl-3-methylimidazolium thiocyanate [BMIM][SCN] [26], 1-butyl-3-methylimidazolium tosylate [BMIM][TOS] [32], 1-ethyl-3-methylimidazolim ethylsulfate [EMIM][EtSO_4_] [33,34], and for ammonium-based ionic liquids, *i.e*., for didecyldimethylammonium nitrate [DDA][NO_3_] [35], or for ethyl-(2-hydroxyethyl)-dimethyl-ammonium tetrafluoroborate C_2_BF_4_ [36]. Water, being a polar solvent, can interact with the polar anion as [EtSO_4_]^−^ or [NO_3_]^−^, but on the other side the long alkane chains as (C_10_) of [DDA]^+^ cation are hydrophobic, hence there is little interaction of water with cation, thus mainly the anion entity is favoured in the {IL + water} mixtures.

Mixtures of {IL + an alcohol} have been discussed in many papers and can reveal the complete miscibility in the liquid phase (liquid at room temperature ILs) [33]; the simple eutectic mixtures with complete miscibility in the liquid phase as the ILs measured in this work and for example [BMIM][TOS] [32], or [DDA][NO_3_] [35]. They may reveal the SLE phase diagrams with the immiscibility gap in the liquid phase [36]. However, the most popular is the LLE phase diagram with the upper critical solution temperature (UCST) [37–42]. An interesting analysis of LLE in the mixtures of imidazolium-based ILs with an alcohol and water has shown the most important factors that govern the phase behaviour of ionic liquids with these solvents [37,38]. A systematic decrease in solubility was observed with an increase of the alkyl chain length of an alcohol [37–42].

## 3. Modelling

Since the solid-solid phase transitions were observed in our ILs, and the change of heat capacity at the melting temperature was assumed to be Δ_fus_*C*p = Δ_fus_*H/T*_fus_, a general thermodynamic equation relating temperature, *T*^SLE^ and the mole fraction of the IL, *x*_1_ in the water and in the 1-alcohols have been fitted to all the sets of experimental SLE data [43]:

(1) $$-\text{ln}\;x_{1}=\frac{\Delta_{\text{fus}}H}{R}\left(\frac{1}{T}-\frac{1}{T_{\text{fus}}}\right)+\frac{\Delta_{\text{tr}}H}{R}\left(\frac{1}{T}-\frac{1}{T_{\text{tr}}}\right)-\frac{\Delta_{\text{fus}}C_{p}}{R}\left(\text{ln}\frac{T}{T_{\text{fus}}}+\frac{T_{\text{fus}}}{T}-1\right)+\text{ln}\;\gamma_{1}$$

where *x*_1_, *γ*_1_, Δ_fus_*H*, Δ_fus_*C*p, *T*_fus_, *T*, Δ_tr_*H* and *T*_tr_ are mole fraction, activity coefficient, enthalpy of fusion, difference in solute heat capacity between the liquid and solid phase at melting temperature, melting temperature, equilibrium temperature, enthalpy of the solid-solid phase transition and transition temperature, respectively. If a solid-solid phase transition occurs before fusion, the solubility equation for temperatures below that of the phase transition must include the effect of the transition. In this work the (solid-solid) phase transition was far below the experimental points. The enthalpy of melting is assumed to be temperature independent, whereas the activity coefficient is temperature as well as solubility dependent.

In this work three equations were used to describe the experimental data: the Wilson [44], UNIQUAC [45] and the NRTL equation proposed by Renon and Prausnitz [46]. For the ILs used in this work, the molar volumes for the hypothetical subcooled liquids were calculated by the group contribution method described by Barton [30]. The equations used have two adjustable parameters *P*_1_ and *P*_2_ (and the parameter *α* which is fixed, additionally for the NRTL eqn.), which are determined by minimization of the objective function F(*P*_1_, *P*_2_), defined as follows:

(2) $$\text{F}\left(P_{1},P_{2}\right)={\sum_{i=1}^{n}\;\left[T_{\text{exp},i}-T_{\text{calc},i}\;\left(x_{i},P_{1},P_{2}\right)\right]}^{2}$$

where *n* denotes the number of experimental points. The Marquardt algorithm for solving non-linear least squares problems was successfully used in this work. As a measure of the reliability of the correlations, the root-mean-square deviation of temperature, *σ*_T_, has been calculated according to the following definition:

(3) $$\sigma_{\text{T}}={\left\{\sum_{i=1}^{n}\frac{{\left(T_{\text{exp},i}-T_{\text{calc},i}\right)}^{2}}{n-2}\right\}}^{1/2}$$

The values of the parameters and the corresponding root-mean-square deviations of temperature, σ_T_, are reported in Table 5 and the resulting curves are presented together with the experimental points in Figures 2 and 3 for {IL + water} and {[PMPYR][CF_3_SO_3_] + an alcohol} binary systems, respectively.

The results obtained indicate that the equations used were appropriate for providing a reliable description of the SLE measured in this work. The average value of the root-mean-square deviations of temperature, *σ*_T_, for the best NRTL equation was 1.78 K. In this work, the value of parameter *α*, a constant of proportionality similar to the nonrandomness constant of the NRTL equation, had different values in the calculations for different binary systems (*α* = 0.1, *α* = 0.2, *α* = 0.3, *α* = 0.4, *α* = 0.5) to obtain the best correlation.

All the mixtures investigated in this work, show positive or negative deviations from ideality. The differences from ideality were not significantly higher, or lower than one and the values of activity coefficients of ILs in the saturated solutions ranged from 0.62 to 30. It is generally known that for small solubility (*i.e.*, [PMPYR][CF_3_SO_3_] in 1-decanol), the activity coefficients are higher than one.

## 4. Experimental Section

### 4.1. Materials

The IL investigated here, 1-ethyl-1-methylpyrrolidinium triflate, [EMPYR][CF_3_SO_3_] and 1-propyl-1-methylpyrrolidinium triflate [PMPYR][CF_3_SO_3_], with a purity of >99 mass fraction, were purchased from Liquids Technologies (iolitec GmbH& Co. KG, Denzlingen, Germany). Water for the solubility measurements was twice distilled, degassed, deionized and filtered with Milipore Elix 3. The alcohols used in phase equilibria measurements were purchased from Sigma Aldrich Chemie GmbH (Steinheim, Germany). All the alcohols were fractionally distilled in order to achieve a mass percent purity (checked by using gas chromatography) of >99.8% and then stored over freshly activated molecular sieve (type 4Å from Union Carbide). The water content of ILs and the solvents was determined by the Karl-Fisher titration (method TitroLine KF). Samples of the IL and the solvents were dissolved in dry methanol and titrated in 2.5 μL steps. The analysis showed that the water mass fraction in the pure IL, in solvents and in the mixtures with the ionic liquid, was <270 × 10^−6^.

### 4.2. Differential Scanning Microcalorimetry

The temperature of fusion and (solid-solid) phase transition, enthalpy of fusion and (solid-solid) phase transition have been measured using a differential scanning microcalorimetry technique (DSC). The applied scan rate was 5 K·min^−1^, with a power and recorder sensitivities of 16 mJ·s^−1^ and 5 mV, respectively. The apparatus (Thermal Analysis Q200, USA with Liquid Nitrogen Cooling System) was calibrated with a 0.999999 mol fraction purity indium sample. The average value of the melting temperature was (*T*_fus_± 0.1) K (average over three scans). The repeatability of that value was ±0.1 K. The enthalpy of fusion was (Δ_fus_*H*± 0.1) kJ·mol^−1^ and that of (solid-solid) phase transition was (Δ_tr_*H*± 0.1) kJ·mol^−1^. The new data are listed in Table 2.

### 4.3. Apparatus and Experimental Procedure

(Solid + liquid) phase equilibria measurements were carried out using a visual method, according to procedures described previously [47]. Mixtures of the IL + solvent were prepared by weighing the pure components within an accuracy of 1 × 10^−4^ g. The sample was heated very slowly (at less than 2 K h^−1^) with continuous stirring inside a Pyrex glass cell placed in thermostated water bath. The temperature of the disappearance of the solid phase (detected visually) was measured with a calibrated Gallenkamp Autotherm II thermometer totally immersed in the water bath. The uncertainty of temperature and compositions (mole fractions) measurements was ±0.1 K and ±0.0005, respectively. The reproducibility of the measured saturated phase boundary temperatures was better than 0.1 K.

The solubility of the (IL + solvent) mixtures was studied in most of the systems over the whole range of concentration. In the case of alcohols only the ionic liquid rich mole fraction liquidus curves were measured. The eutectic points in the systems with alcohols were not detected because of the very low temperature.

## 5. Conclusions

(Solid + liquid) phase equilibria have been measured in binary systems containing 1-alkyl-1-methylpyrrolidinium triflate ionic liquid and water, or an alcohol. The results obtained indicate that the structure of the pyrrolidinium cation (including the cation’s symmetry and size, *i.e.*, the alkyl substituents and their length) can influence the melting temperature and the phase behaviour of these ionic liquids. The ionic liquid with the more asymmetric cation h([PMPYR][CF_3_SO_3_]) as the lower melting temperature and higher solubility in water and in alcohols. In the case of alcohols the influence is small and seems to be more significant at higher temperatures. Pyrrolidinium-based ILs are highly ordered hydrogen-bonded substances that may interact strongly with water and an alcohol revealing complete miscibility in the liquid phase. The specific interaction of the IL with an alcohol (association between the oxygen of the anion and the hydroxyl group of an alcohol) increases solubility in low molecular weight alcohols as shown in Figure 3. The pyrrolidinium-based ILs are novel compounds which should find new applications in the selective separation of liquids. The results of the correlation with the two parameters NRTL equation displayed acceptable standard deviation.

## Figures and Tables

**Table 1.** Abbreviations, names and structures of investigated ionic liquids.

**Abbreviation**	**Name**	**Structure**
[EMPYR][CF_3_SO_3_]	1-ethyl-1-methylpyrrolidinium triflate	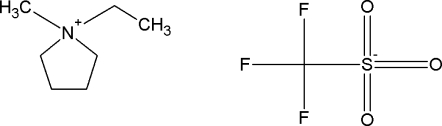
[PMPYR][CF_3_SO_3_]	1-propyl-1-methylpyrrolidinium triflate	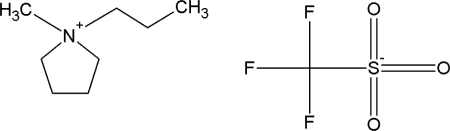

**Table 2.** Thermophysical constants of pure ILs (DSC data).

**Ionic liquid**	***M*/g·mol^−1^**	***V***_**m**_**^298.15^/cm^3^·mol^−1^**	***T***_**fus**_**/K**	**Δ**_**fus**_***H*/kJ·mol^−1^**	***T***_**tr**_**/K**	**Δ**_**tr**_***H*/kJ·mol^−1^**	**Δ**_**fus**_***C*p^a^/J·mol^−1^·K^−1^**
[EMPYR][CF_3_SO_3_]	263.26	239.6^b^	378.5(SLE) 378.4(DSC)	9.99 ± 0.05	234.15	13.03 ± 0.05	0.06^c^
[PMPYR][CF_3_SO_3_]	277.29	255.7^b^	350.7	37.24 ± 0.05	205.15	1.92 ± 0.05	106.2

a Δ_fus_*C*p from the assumption: Δ_fus_*C*p *=* Δ_fus_*H/ T*_fus_;

b calculated as for a hypothetical sub-cooled liquid by the group contribution method described by Barton [30];

c assuming the Δ_fus_*H* = Δ_fus_*H* + Δ_tr_*H*.

**Table 3.** Experimental phase equilibrium temperatures, *T*^SLE^, for {[EMPYR][CF_3_SO_3_] + solvent} binary systems: *x*_1_, mole fraction; *γ*_1_, experimental activity coefficient of the IL.

***x***_**1**_	***T*^SLE^/K**	***γ***_**1**_	***x***_**1**_	***T*^SLE^/K**	***γ***_**1**_
Water					

0.0000	273.2	1.00	0.2087	305.3	2.24
0.0160	271.5	1.07	0.2159	308.4	2.25
0.0252	269.7	1.15	0.2258	311.8	2.25
0.0299	268.0	1.23	0.2308	314.2	2.26
0.0356	266.4	1.28	0.2384	317.5	2.28
0.0425	264.0	1.35	0.2506	322.6	2.30
0.0519	259.7	1.41	0.2555	325.3	2.33
0.0838	242.5	2.01	0.2623	328.0	2.34
0.0903	247.8	2.07	0.2866	333.4	2.27
0.0974	252.7	2.11	0.3055	337.7	2.23
0.1030	256.9	2.16	0.3166	340.4	2.21
0.1083	260.7	2.20	0.3278	344.6	2.23
0.1137	264.0	2.22	0.3480	348.1	2.18
0.1202	267.9	2.24	0.3736	350.9	2.09
0.1267	271.7	2.27	0.4064	355.0	1.99
0.1300	273.2	2.26	0.4262	357.0	1.94
0.1342	275.1	2.26	0.4405	358.6	1.90
0.1418	278.4	2.25	0.4751	362.0	1.82
0.1485	281.6	2.26	0.5224	365.2	1.71
0.1565	285.1	2.26	0.5595	367.3	1.62
0.1652	289.1	2.27	0.5977	368.6	1.54
0.1795	294.4	2.25	0.6521	370.9	1.44
0.1860	297.0	2.25	0.7078	372.9	1.35
0.1905	298.9	2.25	0.8015	376.3	1.22
0.2015	302.9	2.25	1.0000	378.5	1.00

1-Butanol					

0.7388	351.5	1.06	0.8713	366.3	1.03
0.7620	353.7	1.05	0.8885	368.4	1.03
0.7839	355.9	1.04	0.9125	370.6	1.02
0.8135	359.0	1.03	0.9430	373.2	1.01
0.8368	361.6	1.03	0.9624	375.3	1.01
0.8529	363.8	1.03	1.0000	378.5	1.00

**Table 4.** Experimental phase equilibrium temperatures, *T*^SLE^, for {[PMPYR][CF_3_SO_3_] + solvent} binary systems: *x*_1_, mole fraction; *γ*_1_, experimental activity coefficient of the IL.

***x***_**1**_	***T*^SLE^/K**	***γ***_**1**_	***x***_**1**_	***T*^SLE^/K**	***γ***_**1**_
Water					

0.0000	273.2	1.00	0.3991	301.5	0.31
0.0189	272.2	0.97	0.4287	304.6	0.34
0.0451	270.7	0.95	0.4543	309.0	0.39
0.0775	268.8	0.90	0.4724	310.6	0.41
0.0788	267.9	0.87	0.4901	312.3	0.42
0.1044	271.5	0.23	0.5180	315.2	0.46
0.1209	273.5	0.22	0.5570	319.8	0.52
0.1427	277.0	0.23	0.5934	323.6	0.58
0.1978	280.9	0.21	0.6470	328.3	0.65
0.2385	284.9	0.22	0.6708	330.9	0.69
0.2813	289.5	0.24	0.7069	334.1	0.75
0.2932	291.2	0.25	0.8461	344.0	0.92
0.3138	293.3	0.26	0.8775	346.0	0.96
0.3591	297.4	0.28	1.0000	350.7	1.00
0.3810	299.7	0.30			

1-Butanol					

0.0698	295.6	1.32	0.4554	320.4	0.66
0.0896	296.8	1.10	0.4827	322.0	0.66
0.1041	297.7	0.99	0.5071	323.5	0.67
0.1119	298.3	0.95	0.5417	325.6	0.69
0.1309	299.4	0.86	0.5698	327.7	0.72
0.1528	300.8	0.79	0.5922	329.1	0.73
0.1735	302.1	0.74	0.6202	330.6	0.74
0.2050	304.0	0.69	0.6444	331.9	0.75
0.2384	306.2	0.66	0.6776	334.2	0.79
0.2681	307.9	0.63	0.7160	336.5	0.81
0.2897	309.4	0.63	0.7585	338.8	0.84
0.3117	310.9	0.63	0.7962	340.6	0.86
0.3330	312.2	0.62	0.8372	342.4	0.88
0.3535	313.4	0.62	0.8850	344.3	0.89
0.3716	314.8	0.63	0.9284	346.4	0.92
0.3961	316.4	0.63	0.9570	348.1	0.95
0.4206	318.0	0.64	1.0000	350.7	1.00

1-Hexanol					

0.0863	310.4	2.21	0.5027	328.8	0.85
0.1164	312.2	1.78	0.5298	329.9	0.84
0.1457	313.8	1.53	0.5633	331.0	0.83
0.1736	315.2	1.37	0.5965	332.4	0.83
0.2026	316.6	1.25	0.6343	333.8	0.83
0.2344	318.0	1.15	0.6781	335.5	0.83
0.2595	319.1	1.09	0.7085	336.8	0.83
0.2886	320.2	1.03	0.7544	338.6	0.84
0.3196	321.3	0.97	0.7954	340.5	0.86
0.3586	322.8	0.92	0.8482	342.7	0.87
0.3796	323.7	0.91	0.8946	344.8	0.90
0.4207	325.2	0.87	0.9474	347.1	0.92
0.4519	326.4	0.85	1.0000	350.7	1.00
0.4800	327.7	0.85			

1-Octanol					

0.0253	318.1	10.67	0.3984	334.4	1.35
0.0344	320.0	8.53	0.4457	335.2	1.24
0.0456	321.7	6.93	0.5018	336.2	1.15
0.0557	323.2	6.05	0.5409	337.1	1.10
0.0676	324.3	5.23	0.5787	337.9	1.07
0.0867	325.5	4.29	0.6299	338.8	1.01
0.1175	327.2	3.40	0.6673	339.6	0.99
0.1580	328.9	2.71	0.7047	340.2	0.96
0.1666	329.3	2.62	0.7514	341.3	0.94
0.1980	330.2	2.28	0.7962	342.4	0.92
0.2396	331.1	1.96	0.8445	343.8	0.92
0.2809	332.1	1.74	0.8986	345.8	0.93
0.3151	332.8	1.60	0.9386	347.5	0.95
0.3582	333.7	1.46	1.0000	350.7	1.00

1-Decanol					

0.0155	331.4	30.66	0.5131	342.4	1.43
0.0489	333.4	10.54	0.5470	342.4	1.34
0.0689	335.0	7.98	0.5580	342.7	1.33
0.0878	335.6	6.41	0.5915	342.8	1.26
0.1015	336.3	5.70	0.5998	342.7	1.24
0.1223	336.9	4.85	0.6407	343.1	1.18
0.1533	338.0	4.04	0.6798	343.1	1.11
0.1928	338.9	3.32	0.7310	343.5	1.05
0.2324	339.6	2.83	0.7648	343.8	1.01
0.2729	340.2	2.47	0.8094	344.5	0.98
0.3126	340.8	2.21	0.8554	345.3	0.96
0.3532	341.1	1.98	0.8848	346.2	0.96
0.3929	341.4	1.80	0.9445	347.8	0.95
0.4337	341.7	1.65	0.9822	350.2	1.00
0.4738	341.9	1.52	1.0000	350.7	1.00

**Table 5.** Correlation of the (solid + liquid) phase equilibrium data of the {IL + water, or + an alcohol} mixtures by means of Wilson, UNIQUAC and NRTL equations: values of parameters and measures of deviations.

	**Parameters**			**RMSD**		

	**Wilson**	**UNIQUAC**	**NRTL**	**Wilson**	**UNIQUAC**	**NRTL**
Solvent	*λ*_12_ – *λ*_22_	*u*_12_ – *u*_22_	*g*_12_ – *g*_22_		*σ*_T_/(K)	
	*λ*_21_ – *λ*_11_	*u*_21_ – *u*_11_	*g*_21_ – *g*_11_			
	J·mol^−1^	J·mol^−1^	J·mol^−1^			

[EMPYR][CF_3_SO_3_]						

Water	–6000.99	-	11108.24	5.68	-	1.77^a^
	14643.09	-	–2407.88			
1-Butanol	86142.98	6189.93	9151.76	2.52	0.82	0.77^b^
	2260.38	–3213.90	–4863.26			

[PMPYR][CF_3_SO_3_]						

Water	–11445.59	-	1729.43	2.53	-	2.04^c^
	7593.47	-	–5366.87			
1-Butanol	–4777.45	–2236.81	–6688.84	5.62	5.01	2.45^a^
	3836.80	9586.35	16576.57			
1-Hexanol	59160.06	–1875.88	–10141.14	3.50	3.01	1.97^d^
	–186.86	4414.00	19911.02			
1-Octanol	7114.00	–1005.80	–398.14	1.40	1.41	1.41^c^
	1263.48	2427.37	7364.46			
1-Decanol	9729.67	–797.71	2740.31	1.20	3.01	2.09^e^
	2622.93	2348.27	8010.29			

a non-randomness parameter (*α*_12_ = *α*_21_) assumed to be constant and equal 0.2;

b *α*_12_ = *α*_21_ = 0.3;

c *α*_12_ = *α*_21_ = 0.4;

d = *α*_12_ = *α*_21_ = 0.1;

e *α*_12_ = *α*_21_ = 0.5.
